# Dynamic Behavior of a Precast and Partial Steel Joint under Various Shear Span-to-Depth Ratios

**DOI:** 10.3390/ma14092162

**Published:** 2021-04-23

**Authors:** Guoxi Fan, Jing Yang, Ye Wang, Qiyi Zhang, Jing Jia, Wanpeng Cheng

**Affiliations:** 1 Shandong Province Key Laboratory of Ocean Engineering, College of Engineering, Ocean University of China, Qingdao 266100, China; yangjing2416@163.com (J.Y.); WangYezest@163.com (Y.W.); zhangqiyi@163.com (Q.Z.); jingjia@ouc.edu.cn (J.J.); 2 State Key Laboratory of Coastal and Offshore Engineering, Dalian University of Technology, Dalian 116024, China; chengwp@sina.com

**Keywords:** precast partial steel, beam–column joint, strain rate, shear span-to-depth ratio, dynamic behavior

## Abstract

The dynamic behavior of a PPSRC beam–column joint is related to constraint effect, strength deterioration and strain rate effect. Then, it can be assessed by bearing capacity, stiffness degradation, displacement ductility and energy consumption. The results show that the increased strain rate causes growth in ring stiffness, bearing capacity and energy consumption of PPSRC beam–column joints. However, the influence of shear span-to-depth ratio on dynamic mechanical properties of PPSRC beam–column joints is more obvious than that of strain rate. Regardless of strain rate, the bearing capacity, initial stiffness, ring stiffness and energy consumption of PPSRC beam–column joints decrease as the shear span-to-depth ratio increases. Moreover, the ring stiffness under reverse direction is smaller than that the under forward direction at each displacement level. However, the stiffness degradation under a lower shear span-to-depth ratio is more obvious than that under a higher shear span-to-depth ratio. Moreover, the displacement ductility with a higher shear span-to-depth ratio is better than that with a lower shear span-to-depth ratio. Finally, the mechanical properties of PPSRC beam–column joints are affected by the extension length of partial steel plate, and the reasonable extension length of the partial steel plate in the column is affected by the shear span-to-depth ratio.

## 1. Introduction

Compared with traditional cast-in situ construction, a precast concrete structure possesses higher business competitiveness due to its environmental protection, economy and excellent mechanical properties [1]. For example, in Turkey, precast concrete structures have been widely used in the construction industry [2]. Furthermore, precast concrete structures possess a bright future in improving the seismic performance of buildings. Yooprasertchai and Warnitchai [3] indicated that the precast hybrid moment-resisting frames showed better ductility and excellent seismic performance compared with traditional cast-in situ reinforced concrete beam–column frames. Therefore, many countries are promoting the use of precast construction under the pressure of the increasing housing demand [4]. When referring to structural members, the beam–column joint may affect the mechanical properties of the overall structure, which is a critical member [5]. Under external loading conditions, the beam–column joint is often first damaged because of its complex stress characteristics. Along with the continuous accumulation and development of damage, the mechanical properties of beam–column joints deteriorate gradually, resulting in final failure, which causes the destruction of overall structure [6,7]. The design of beam–column joints must be fully considered in the seismic design of moment-resisting frames [8]. Several experimental studies indicate that the mechanical properties of beam–column joints have a significant contribution to the overall structure [9]. Post-earthquake reconnaissance efforts have also attributed the collapse of many structures to the destruction of beam–column joints [10]. Beam–column joints are also the key area of inelastic response of a reinforced concrete frame structure under strong seismic loading, which has a significant impact on the seismic response of the reinforced concrete frame structure [11]. Moreover, the treatment of beam–column joints is more difficult in precast structures, in which they are the weakest and most critical part of precast structures [5]. Therefore, designing a beam–column joint in a precast concrete structure is crucial.

Researchers were of great assistance in research into precast beam–column joints or connection. The conclusions were that precast specimens showed slightly reduced mechanical properties, including stiffness and energy consumption [12]. Despite this, a reasonable design can ensure the good working performance of a precast structure. Compared to bearing capacity and ductility performance, it can be found that the mechanical properties of a precast structure nearly matched those of a monolithic structure [13]. Through the reasonable design of beam–column joints, the precast structure showed comparable mechanical behavior to the monolithic structure [14]. Consequently, design guides of monolithic structures can be used to design the precast structure [15].

Based on the difference in the connection method, precast concrete members can be connected at the site by wet connection or dry connection [16,17]. The wet connection is generally filled with cast-in-place concrete or high-grade grouting, while the dry connection is riveted or welded at the joint by bolts, metal plates and other components. In general, the mechanical properties of wet connections are superior to those of dry connections [4]. The wet connection shows more fixity, while the dry connection shows discontinuity and poor constraint, which would weaken the catenary action of precast structure [18]. In these circumstances, the precast structure possesses insufficient constraints, which is more likely to lead to progressive collapse. When referring to the wet connection, cast-in-place concrete is required to connect various precast construction components. The casting operation is affected by the climate and the technical level of workers, so the connection quality is not easily controlled.

With these considerations in mind, a new type of beam–column joint, i.e., precast and a partial steel reinforced concrete (PPSRC) beam–column joint was proposed for a precast concrete structure. To evaluate the feasibility of the PPSRC beam–column joint, cyclic loading tests were performed by authors in a previous paper to study the mechanical behavior of the PPSRC beam–column joint [19]. Compared with the RC beam–column joint, the PPSRC beam–column joint exhibited higher bearing capacity, better ductility performance and more energy consumption under low-frequency cyclic loading. Unfortunately, the column was first destroyed due to the insufficient extension length of the partial steel plate in the column. According to the existing specifications [20], the column should not be destroyed before the beam is destroyed. Therefore, further research on the mechanical properties of the PPSRC beam–column joint needs to be carried out.

On the other hand, multiple factors may affect the mechanical properties of the PPSRC beam–column joint, including the shear span-to-depth ratio, sectional characteristics, reinforcement detailing, concrete grade, the axial compression ratio, reinforcement anchorage type, and joint shear stress [21,22]. Among these, the shear span-to-depth ratio is an important factor in affecting the mechanical properties of the PPSRC beam–column joint. The effect the of shear span-to-depth ratio on the mechanical properties of RC members has been studied by many researchers. Bousselham and Chaallal [23,24] studied the shear contribution for strengthened beams. When the shear span-to-depth ratio was higher, the shear contribution supplied by FRP was larger. Li and Leung [25] studied the mechanical behavior of strengthened beam members. A similar conclusion can be drawn that the shear span-to-depth ratio significantly influenced the shear contribution supplied by FRP. Shear strengths for the RC beam contributed by concrete and stirrup reinforcement were also discussed. The following conclusions can be drawn that shear strength contributed by concrete decreased sharply when the shear span-to-depth ratio increased, and an opposite trend can be obtained for shear strength contributed by stirrup reinforcement [26]. The effect of the shear span-to-depth ratio on the bending moment of fifteen specimens was tested. Research showed that the ultimate moment increased with the increase in the shear span-to-depth ratio [27].

Although a lot of research has been carried out to evaluate the effect of the shear span-to-depth ratio on the mechanical properties of RC members, the effect of shear span-to-depth ratio on the mechanical behavior of the PPSRC beam–column joint has been less investigated. Additionally, a comprehensive investigation on the dynamic behavior of the PPSRC beam–column joint under various shear span-to-depth ratios has not yet been carried out. The effect of the shear span-to-depth ratio on the dynamic behavior of the PPSRC beam–column joint is hence focused upon in this paper. Generally, experimental study is more suitable to assess the dynamic behavior of the PPSRC beam–column joint, but it is hard to realize in many cases. In this case, the finite element analysis is more suitable. Considered the constraint effect, strength deterioration and strain rate effect, a finite element model was first developed to assess dynamic behavior of the PPSRC beam–column joint. After a description of the experimental overview, the rationality of the finite element model was studied. The validated finite element model was then adopted to assess the dynamic behavior of the PPSRC beam–column joint. Finally, recommendations were made to design the reasonable extension length the of partial steel plate under various shear span-to-depth ratios.

## 2. Finite Element Model

### 2.1. Fundamental Modifications

The finite element model was developed to the assess dynamic behavior of the PPSRC beam–column joint, where the characteristics of the cross-section, the reinforcement bars and steel plates properties are the same as that of specimens mentioned in a previous paper [19]. The numerical analysis will be performed by employing the software ABAQUS. Before establishing the finite element model, the following three problems should be considered.

(1) Under the influence of Poisson’s ratio, concrete will expand laterally under compression. However, the expansion deformation of concrete will be prevented due to the existence of stirrup reinforcements and steel plates, which have a certain constraint effect [28]. If only the constraint effect of stirrup reinforcements on concrete is considered, the theoretical and numerical results are not in good agreement [29,30]. Therefore, in order to reasonably represent the actual mechanical properties of members, the constraint effect of steel plates should also be considered. Based on the above analysis, the constraint effect of stirrup reinforcements and steel plates should be considered to ensure the accuracy of numerical analysis.
(2) At present, the software ABAQUS is adopted by many scholars to simulate the mechanical properties of beam–column joints under cyclic loading. However, no descending segment existed in the obtained hysteretic curves [31,32]. As a result, it is impossible to reflect whether the beam–column joint is invalid through the hysteretic curves. The reason for the above phenomenon is that the strength degradation of the material is not considered in the selected material constitutive model when the numerical analysis is implemented. To solve the above problem, the descending segment of skeleton curve is introduced into the steel constitutive model according to the published papers [33]. Although the accumulative damage fails to be considered, the strength degradation and failure behavior of steel can be described through this method.
(3) Concrete and steels are both rate-sensitive materials, which will exhibit various mechanical behaviors with conditions of various strain rates. The PPSRC beam–column joint is constituted of concrete and steel, so it is also rate-sensitive. When subjected to dynamic loadings, the response characteristics and damage mechanism of the PPSRC beam–column joint will show obvious differences from that subjected to static or quasi-static loadings [34]. However, knowledge of the strain rate effect on the dynamic behavior of the PPSRC beam–column joint is limited, with emphasis primarily placed on the observed material dynamic mechanical properties and less attention paid to changes in the dynamic behavior of the PPSRC beam–column joint. In view of this, it is of great significance to consider the strain rate effect in the finite element analysis.

### 2.2. Improved Material Constitutive Models

#### 2.2.1. Constraint Effect

The plastic damage model in the software ABAQUS may be adopted to simulate the mechanical properties of members under monotonic loading or cyclic loading. As mentioned above, the constraint effect should be considered due to the existence of stirrup reinforcements and steel plates. With this in mind, the stress–strain constitutive relationship for concrete in the plastic damage model will be established with the consideration of the constraint effect induced by stirrup reinforcements and steel plates. Extensive and mature research on the constraint mechanism of stirrup reinforcements and steel plates has been conducted by Mander and other scholars. Consequently, the stress–strain constitutive relationship for confined concrete can be established by referring to the published paper [35]. In addition, the stress–strain constitutive relationship mentioned in the standard GB50010-2010 was adopted for unconfined concrete [36]. More details of the standard GB50010-2010 [36] can be found in the following section.

For the confined concrete, the key to establish the stress–strain constitutive relationship is to determine the effective lateral restraint stress and increasing factor of strength (*K*). The formula for calculating the concrete stress (*σ*) in the Mander model is given by Reference [35]. The published paper [29] will be adopted to calculate the increasing factor of strength (*K*). Before the stress–strain constitutive relationship for confined concrete is obtained, another problem is to obtain the effective lateral restraint stress according to the confined region. In light of the simplified restraint state of confined concrete, it can be divided into high restraint concrete, weak restraint concrete and unconfined concrete. Thus, the increasing factor of strength (*K*) should be calculated separately for different regional partitions, and the confined concrete in this paper can be divided into three types.

(1) Partially restraint concrete (PRC) surrounded by I-steel flanges of the beam. The effective lateral restraint stress of the PRC is the linear superposition of restraint stress provided through steel flanges and stirrup reinforcements. In addition, the effective lateral restraint stress on the PRC can be given by Reference [29].
(2) Highly restraint concrete (HRC) and partially restraint concrete (PRC) surrounded by cross-shaped steel of the column. The effective lateral restraint stress of the HRC and PRC can be calculated by Reference [29].
(3) Partially confined concrete (PCC) surrounded by the stirrup reinforcements. The effective lateral restraint stress of the PCC provided through stirrup reinforcements of beam and column could be calculated as follows [35].

(1) $$f_{l,x}^{‘}={K^{\prime }}_{e}\rho_{x}f_{x}{}_{,h}$$

(2) $$f_{l,y}^{‘}={K^{\prime }}_{e}\rho_{y}f_{y,h}$$

(3) $$f_{{}_{l,x}}^{‘}=\frac{A_{sx}}{sd_{c}}f_{x,h}=\rho_{x}f_{x,h}$$

(4) $$f_{l,y}^{‘}=\frac{A_{sy}}{sb_{c}}f_{yh}=\rho_{y}f_{y,h}$$

(5) $$K_{e}^{’}=\frac{\left(1-\sum_{i=1}^{n}\frac{{\left(w_{i}^{’}\right)}^{2}}{6b_{c}d_{c}}\right)\left(1-\frac{s^{’}}{2b_{c}}\right)\left(1-\frac{s^{’}}{2b_{c}}\right)}{\left(1-\rho_{cc}\right)}$$

where $K_{e}^{’}$ is the effective restraint coefficient of stirrup reinforcements; $f_{l,x}^{‘}$ and $f_{l,y}^{‘}$ are the effective restraint stresses in the *x*-direction and *y*-direction, respectively; *ρ_cc_* is the longitudinal reinforcement ratio; *b_c_* is effective section width in *x* direction; *d_c_* is the effective section width in *y* direction; $w^{\prime }i$ is the clear spacing of the adjacent longitudinal reinforcements; $s^{\prime }$ is the vertical clear spacing of the spiral reinforcements or hooped reinforcements; *ρ_x_* and *ρ_y_* represent the reinforcement ratios of the rectangular reinforced concrete member in the horizontal and vertical directions, respectively; *f_x,h_* and *f_y,h_* are yielding strengths supplied by horizontal and vertical reinforcement bars, respectively.

For the unconfined concrete (UC), the stress–strain constitutive relationship mentioned in standard GB50010-2010 is adopted [36]. The expressions of compression constitutive laws for concrete are as follows:

(6) $$\sigma_{c}=\left(1-d_{c}^{\prime }\right)E_{c}\varepsilon_{c}$$

(7) $$d_{c}^{\prime }=\left\{{}_{1-\frac{\rho_{c}}{\alpha_{c}{\left(x_{c}-1\right)}^{2}+x_{c}},\;x_{c}\;>\;1}^{1-\frac{\rho_{c}n}{n-1+x_{c}{}^{n}}\;,\;x_{c}\;\leq \;1}\right.$$

(8) $$x_{c}=\frac{\varepsilon_{c}}{\varepsilon_{c,r}},\;\rho_{c}=\frac{f_{c,r}}{E_{c}\varepsilon_{c,r}},\;n=\frac{E_{c}\varepsilon_{c,r}}{E_{c}\varepsilon_{c,r}-f_{c,r}}$$

where *σ_c_* and *ɛ_c_* are stress and strain under compression, respectively; *E_c_* is the elastic modulus for unconfined concrete; $d_{c}^{\prime }$ is damage evolution coefficient of concrete under uniaxial compression; *α_c_* is coefficient corresponding to descending branch of the concrete stress–strain curve under uniaxial compression; *f_c,r_* is a typical value corresponding to concrete strength under uniaxial compression; *ε_c,r_* is compressive peak strain corresponding to the typical value of uniaxial compression strength *f_c,r_*; *ρ_c_*, *x_c_* and *n* are calculation parameters.

Following the provisions of the code GB50010-2010 [36], the expressions of tension constitutive laws for concrete are as follows.

(9) $$\sigma_{t}=\left(1-d_{t}\right)E_{c}\varepsilon_{t}$$

(10) $$d_{t}=\left\{{}_{1-\frac{\rho_{t}}{\alpha_{t}{\left(x_{t}-1\right)}^{1.7}+x_{t}},\;x_{t}\;>\;1}^{\begin{array}{l} 1-\rho_{t}\left[1.2-0.2x_{t}^{5}\right]\;,\;x_{t}\;\leq \;1 \end{array}}\right.$$

(11) $$x_{t}=\frac{\varepsilon_{t}}{\varepsilon_{t,r}},\;\rho_{t}=\frac{f_{t,r}}{E_{c}\varepsilon_{t,r}}$$

where *σ_t_* and *ɛ_t_* are stress and strain under tension, respectively; *d_t_* is damage evolution coefficient for concrete under uniaxial tension; *α_t_* is a parameter value corresponding to the descending branch of the concrete stress–strain curve under uniaxial tension; *f_t,r_* is a typical value corresponding to concrete strength under uniaxial tension; *ε_t,r_* is tensile peak strain corresponding to the typical value of uniaxial tension strength *f_t,r_*; *ρ_t_* and *x_t_* are calculation parameters.

**Figure 1:**
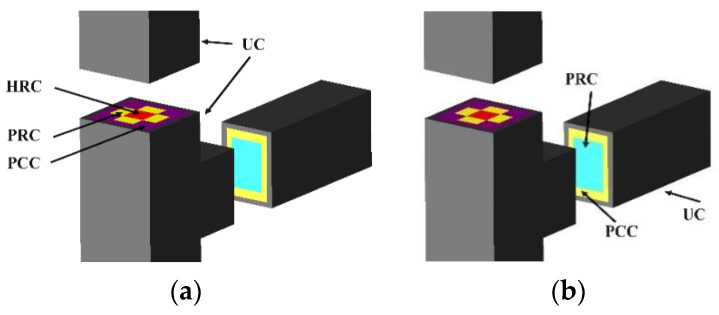
Confined concrete in different regions: (**a**) concrete in the column; (**b**) concrete in the beam.

For the PPSRC beam–column joint, the concrete in the column can be divided into four types, i.e., HRC, PRC, PCC and UC, while the concrete in the beam can be divided into three types, i.e., PRC, PCC and UC, which can be found from Figure 1.

**Figure 2:**
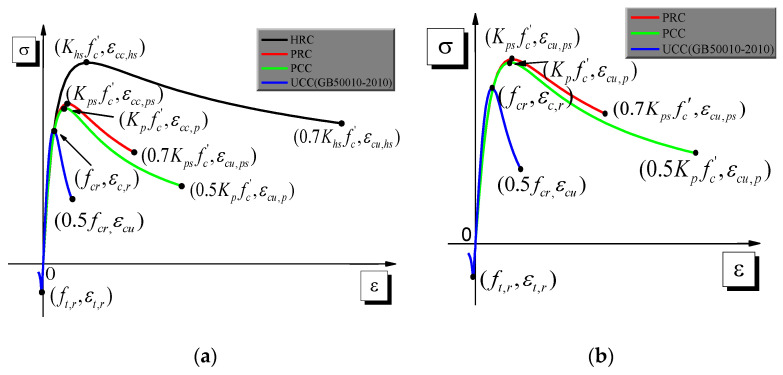
The stress–strain constitutive relationship of different regional partitions: (**a**) concrete in the column; (**b**) concrete in the beam.

After the previously mentioned adjustments were made, the concrete stress–strain constitutive relationship of different regional partitions could be obtained based on the existing literature [29]. The stress–strain constitutive relationship of HRC, PRC, PCC, and UC are presented in Figure 2, where $\varepsilon_{cc,hs}$, $\varepsilon_{cc,ps}$, $\varepsilon_{cc,p}$, $\varepsilon_{c,r}$ are compressive strains corresponding to the concrete compressive strength of HRC, PRC, PCC, and UC. The ultimate strains of HRC and PRC, i.e., $\varepsilon_{cu,hs}$ and $\varepsilon_{cu,ps}$, are assumed to correspond to $0.7K_{hs}f_{c}^{’}$ and $0.7K_{ps}f_{c}^{’}$, while the ultimate strains of PCC and UC, i.e., $\varepsilon_{cu,p}$ and $\varepsilon_{cu}$, are assumed to correspond to $0.5K_{p}f_{c}^{’}$ and $0.5f_{cr}$ [29].

Other material parameters of concrete including density *ρ*, Poisson ratio *μ*, shear dilation angle *Ψ*, flow potential eccentricity *Ε’*, ultimate stress ratio of biaxial compression to uniaxial compression *α_f_*, the second stress invariant ratio of the compression meridian plane to tension meridian plane *K*_1_, viscosity parameter *ν*, can be found from Table 1.

#### 2.2.2. Strength Deterioration

**Figure 3:**
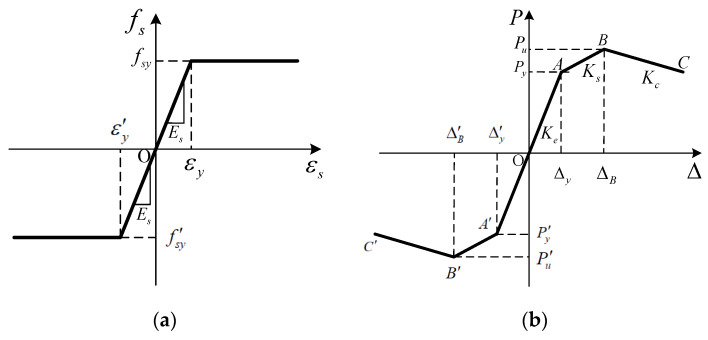
Stress–strain constitutive models: (**a**) reinforcement bar; (**b**) steel plate.

Since the amount of steel plates in the PPSRC beam–column joint core area is more than that of reinforcement bars, the stirrup reinforcements and longitudinal reinforcement bars of the PPSRC beam–column joint are assumed to be ideal elastoplastic materials that can simplify numerical computation. Before reinforcement bar yielding, the stress–strain is a diagonal line. After reinforcement bar yielding, the stress–strain relationship keeps a horizontal line, which can be found in Figure 3a. As previously assumed, the strength degradation should be considered in the stress–strain constitutive model for steel plates. Therefore, a trilinear model with descending segment is adopted to describe the stress–strain constitutive relationship for steel plates [33], as shown in Figure 3b. Among them, OA represents the elastic stage, AB represents post-yielding stage, BC represents descending stage. In addition, *K_e_*, *K_s_* and *K_c_* represent the stiffness of the elastic stage, post-yielding stage and descending stage, respectively. The calculation method of the above stiffness is given by Reference [33]. Moreover, *P_y_* and Δ*_y_* represent the yielding strength and the yielding displacement, while Δ*_B_* represents the peak displacement corresponding to the ultimate strength *P_u_* (*P_y_* = 0.8*P_u_*) [37].

#### 2.2.3. Strain Rate Effect

When subjected to dynamic loadings, the response characteristics and damage mechanism of the PPSRC beam–column joint will show obvious differences from that subjected to static or quasi-static loadings, so it is important to understand the dynamic behavior of the PPSRC beam–column joint. The basic reason lies in the strain rate effect that the mechanical behavior of structural materials changes as the strain rate changes. Thus, reasonable consideration should be given to the strain rate effect before establishing material stress–strain constitutive models. The aforementioned strain rate effect will be included in the plastic damage model of concrete, which is described by the following formula. When the concrete is under uniaxial compression, the dynamic increase factor (*DIF_fc_*) is calculated from the following formula [38].

(12) $$DIF_{fc}=\frac{f_{cd}}{f_{cs}}={\left(\frac{\dot{\varepsilon }}{{\dot{\varepsilon }}_{0}}\right)}^{1.026\alpha }\;\dot{\varepsilon }\;\leq \;30/s$$

(13) $$\alpha =\frac{1}{5+9\frac{f_{cs}}{f_{0}}}$$

When the concrete is under uniaxial tension, the dynamic increase factor (*DIF_ft_*) is calculated from the following formula [38].

(14) $$DIF_{ft}=\frac{f_{td}}{f_{ts}}={\left(\frac{\dot{\varepsilon }}{{\dot{\varepsilon }}_{0}}\right)}^{1.026\alpha }\;\dot{\varepsilon }\;\leq \;30/s$$

(15) $$\alpha =\frac{1}{10+6\frac{f_{cs}}{f_{0}}}$$

More details of various parameters in the formulae mentioned above are shown in Reference [38].

The yielding strength, ultimate strength and yielding strain of steel materials under dynamic loadings could be obtained by the dynamic constitutive model, which is proposed by Li and Li [39]. The related formulae are as follows.

(16) $$\frac{f_{yd}}{f_{ys}}=1+c_{f}\mathrm{lg}\frac{\dot{\varepsilon }}{{\dot{\varepsilon }}_{0}}$$

(17) $$c_{f}=0.1709-3.289\times 10^{-4}f_{ys}$$

(18) $$\frac{f_{ud}}{f_{us}}=1+c_{u}\mathrm{lg}\frac{\dot{\varepsilon }}{{\dot{\varepsilon }}_{0}}$$

(19) $$c_{u}=0.02738-2.982\times 10^{-5}f_{ys}$$

(20) $$\frac{\varepsilon_{hd}}{\varepsilon_{hs}}=1+c_{h}\mathrm{lg}\frac{\dot{\varepsilon }}{{\dot{\varepsilon }}_{0}}$$

(21) $$c_{h}=0.9324-0.00212f_{ys}$$

More details of various parameters in the formulae mentioned above are shown in Reference [39].

## 3. Finite Element Model Verification

### 3.1. Experimental Overview

Six specimens (SI1, SI2, SI3, SE1, SE2, SE3) were cast in which the section characteristic, longitudinal reinforcement bars and stirrup reinforcements were the same [19]. The properties of the concrete, longitudinal reinforcement bar, stirrup reinforcement and steel plate are shown in Table 2.

**Figure 4:**
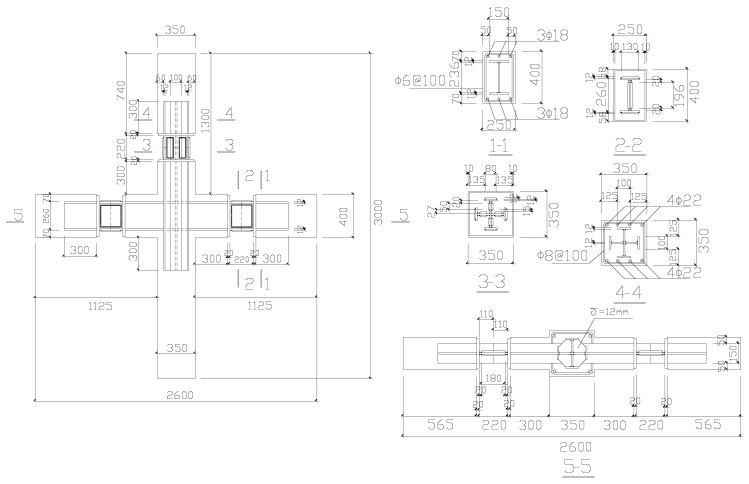
Schematic diagram of reinforcement bars and steel plates of the PPSRC beam–column joint. (Unit: millimeters)

The concrete grade is C30, while the grades of the longitudinal reinforcement bar and stirrup reinforcement are HRB335 and HPB235, respectively. The grade of the steel plate is Q235b for the PPSRC beam–column joint. The selected strength of the materials meets the design requirements specified by GB50011-2010 and GB50010-2010 [20,36]. The layout of the reinforcement bars and steel plates is shown in Figure 4. Cyclic loading tests were carried out. More details about the PPSRC beam–column joint are shown in Reference [19].

**Figure 5:**
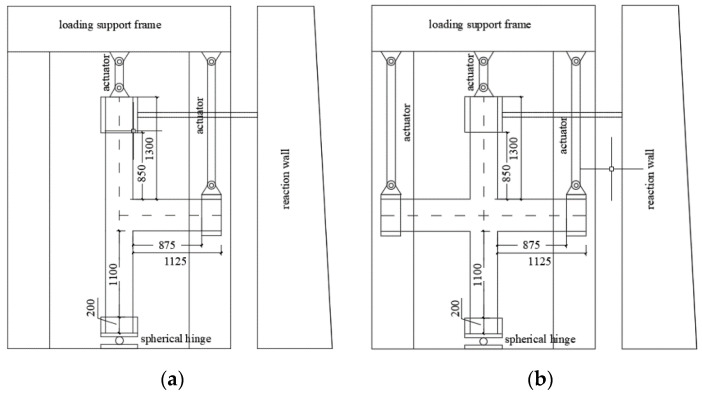
Schematic diagram of the loading apparatus: (**a**) exterior beam–column joint, (**b**) interior beam–column joint.

The loading apparatus of the exterior and interior beam–column joints are shown in Figure 5.

### 3.2. Information of the Finite Element Model

Different finite element models of the PPSRC beam–column joint were established, as shown in Table 3. Finite element models FS2 and FSE1 correspond to specimens SI2 and SE1, respectively.

**Figure 7:**
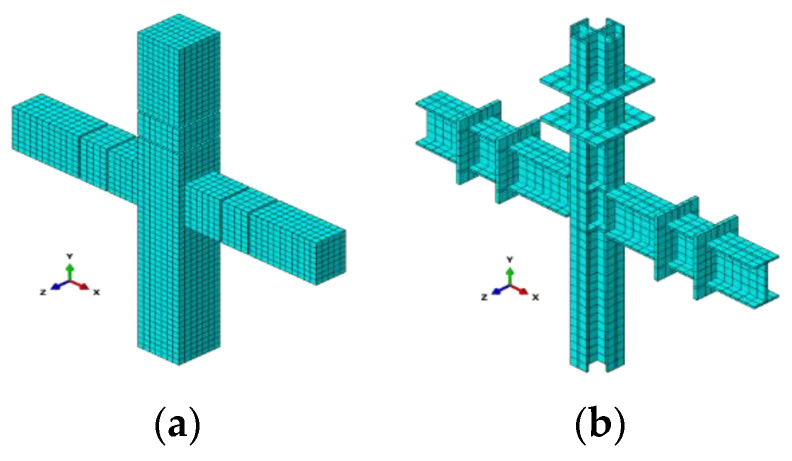
Finite element analysis model: (**a**) mesh dividing for the concrete (**b**) mesh dividing for the steel plate.

Solid element C3D8R was used for the concrete and steel plate, and truss element T3D2 was used for reinforcement. The reinforcement and steel plate were embedded in concrete. The schematic diagram of mesh dividing for the PPSRC beam–column joint is shown in Figure 7. The boundary conditions of the finite element model were set before the numerical analysis. The displacement in the X, Y, Z directions and the rotation angle in the X, Y directions were constrained at the bottom of the column, while the displacement in the X, Z directions and the rotation angle in the XZ, YZ planes were constrained at the top of the column. In addition, the vertical load was applied at the top of the column. Moreover, the displacement in Y direction was only released at the end of the beam.

### 3.3. Verification of the Finite Element Model

**Figure 8:**
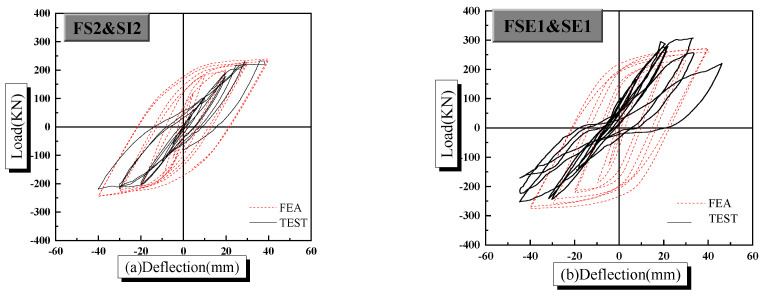
Comparison results of load-deflection hysteretic curves of PPSRC beam–column joints: (**a**) FS2 and SI2; (**b**) FSE1 and SE1.

Comparison results can be found in Figure 8, where the solid line and dotted line represent experimental results and numerical computation results, respectively.

Compared with experimental results, the ultimate bearing capacity and maximum displacement of finite element analysis results are almost consistent. It should be mentioned that the pinching effect of load-deflection hysteretic curves is rarely realized due to tie constraints between elements in the software ABAQUS. The pinching effect is caused by bond slipping; however, the bond slipping is not considered when tie constraints between elements are used. For specimen SE1, the steel plate was rusted before welding. After the test, the specimen SE1 was sectioned. It was found that the weld was destroyed. The above problem was not considered in the finite element analysis, which caused the discrepancy between the numerical solution and the experiment result. In addition, the changing trend of load-deflection hysteretic curves of numerical computation results is consistent when compared with the experimental results, and it illustrates the correctness of proposed finite element model again.

**Figure 9:**
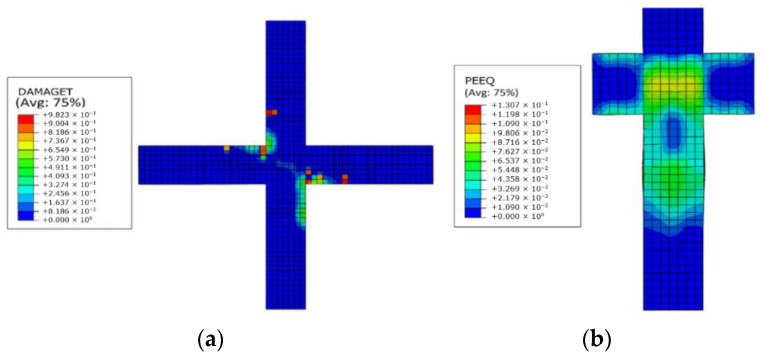
Contours of equivalent plastic strain and concrete tensile damage: (**a**) concrete tensile damage; (**b**) equivalent plastic strain.

For finite element software ABAQUS, the equivalent plastic strain and concrete tensile damage are related to the degree of crack development. Further efforts are made to illustrate the rationality of above-mentioned finite element model, and contours of equivalent plastic strain and concrete tensile damage are also given, which can be found in Figure 9. For an initial stage of finite element analysis, the concrete tensile damage first appears in the following areas that are located at the beam and joint core area. As displacement continues to increase, the equivalent plastic strain of the beam and joint core area continue to increase, while it is mostly concentrated in the core area of the PPSRC beam–column joint and the end of partial steel plate in the column. It is consistent with the experimental phenomena that vertical cracks firstly occur in the beam, and inclined cracks gradually appear along diagonal direction of the joint core area. As displacement continues to increase, cracks that previously appeared in the joint combination are becoming wider and longer. At the same time, new cracks gradually appear in the column. Finally, the concrete at the end of partial steel plate in the column is crushed seriously, but the carrying capacity of the PPSRC beam–column joint does not decrease significantly [19]. Comparison results mentioned above illustrate the effectiveness of the proposed finite element model again.

## 4. Dynamic Behavior of the PPSRC Beam–Column Joint

### 4.1. Finite Element Models

As mentioned earlier, the column was first destroyed due to the insufficient extension length of the partial steel plate in the column. According to the existing specifications [20], the column should not be destroyed before the beam is destroyed. Therefore, a longer extension length of the partial steel plate was selected to obtain better mechanical properties. Further investigations were conducted to explain mechanical properties of the PPSRC joint, and the effect of the shear span-to-depth ratio on dynamic behavior of the PPSRC beam–column joint was analyzed. The basic information of finite element analysis models is given in Table 4.

### 4.2. Hysteretic Curve

**Figure 10:**
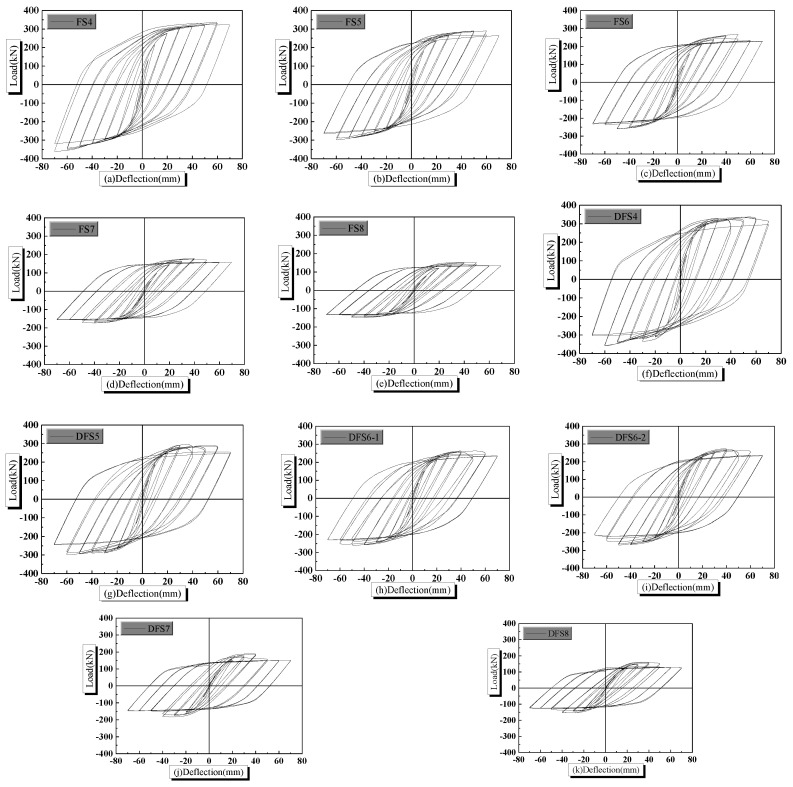
Hysteretic curves for PPSRC joints e: (**a**) model FS4; (**b**) model FS5; (**c**) model FS6; (**d**) model FS7; (**e**) model FS8; (**f**) model DFS4; (**g**) model DFS5; (**h**) model DFS6-1; (**i**) model DFS6-2; (**j**) model DFS7; (**k**) model DFS8.

Analysis results are given in Figure 10. Compared with the actual situation, the tie constraints between elements in the software ABAQUS are ideal. The bond slipping is not considered when tie constraints between elements are used. Therefore, the pinching effect of the load-deflection hysteretic curve is rarely realized. However, it does not affect the changing trend of mechanical properties of the PPSRC joint. Conclusions are drawn from Figure 10 that the descending segment exists in the obtained load-deflection hysteretic curves due to consideration of the strength degradation in the steel plate trilinear model. The aforementioned results of the numerical calculation are more coincident with experimental results, and illustrate the rationality of the improved material constitutive model again.

### 4.3. Skeleton Curve

**Figure 11:**
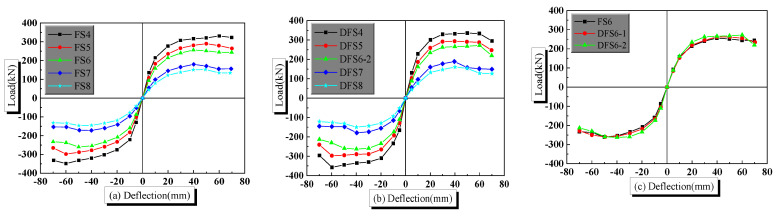
Skeleton curves for PPSRC joints e: (**a**) models FS4, FS5, FS6, FS7 and FS8; (**b**) models DFS4, DFS5, DFS6-2, DFS7 and DFS8; (**c**) models FS6, DFS6-1 and DFS6-2.

The load-deflection skeleton curves of the PPSRC beam–column joint are given in Figure 11. It is not difficult to find the following conclusions that the initial stiffness of the PPSRC beam–column joint decreases as the shear span-to-depth ratio increases. For quasi-static analysis with a strain rate of 1.25 × 10^−5^, taking the average value of forward loading and reverse loading for example, the ultimate bearing capacity of finite element model FS4 (*λ* = 2.3) is 340.0 kN, while the ultimate bearing capacity of finite element model FS8 (*λ* = 4.0) is 149.2 kN. Through compared results, there is a conclusion that the ultimate bearing capacity of the PPSRC joint is reduced by 56.1%. For dynamic analysis with a strain rate of 1.25 × 10^−2^, taking the average value of forward loading and reverse loading for example, the ultimate bearing capacity of finite element model DFS4 (*λ* = 2.3) is 357.6 kN, while the ultimate bearing capacity of finite element model DFS8 (*λ* = 4.0) is 155.1 kN. There is a conclusion that the ultimate bearing capacity of the PPSRC joint is reduced by 56.6%. Regardless of the strain rate, the bearing capacity of the PPSRC beam–column joint decreases as the shear span-to-depth ratio increases. Moreover, another conclusion can be found that both yielding bearing capacity and ultimate bearing capacity of the PPSRC beam–column joint increase as the strain rate increases.

### 4.4. Subsection

Stiffness degradation reflects an accumulation of damage. The ring stiffness can be used to study stiffness degradation of the PPSRC beam–column joint, which is expressed as follows [40].

(22) $$K_{j}^{i}=\sum_{i=1}^{n_{1}}P_{j}^{i}/\sum_{i=1}^{n_{1}}\Delta_{j}^{i}$$

where $K_{j}^{i}$ is ring stiffness; $P_{j}^{i}$ is peak load under *i* cycles, *j* represents the cyclic displacement level; $\Delta_{j}^{i}$ represents the peak displacement under *i* cycles.

**Figure 12:**
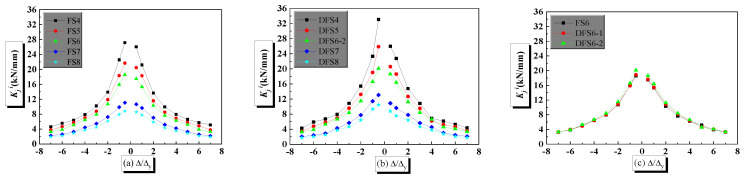
Stiffness degradation curves of PPSRC beam–column joints: (**a**) models FS4, FS5, FS6, FS7 and FS8; (**b**) models DFS4, DFS5, DFS6-2, DFS7 and DFS8; (**c**) models FS6, DFS6-1 and DFS6-2.

Eleven finite element models are analyzed to obtain the stiffness degradation of the PPSRC joint. The first cycle result of cyclic loading is calculated to assess stiffness degradation of the PPSRC joint, and the analysis results are given in Figure 12. For quasi-static analysis with a strain rate of 1.25 × 10^−5^, compared with finite element model FS4 (*λ* = 2.3), ring stiffness of finite element model FS8 (*λ* = 4.0) decreases by 67.5% and 58.7%, when the displacement ductility coefficients (Δ/Δ*_y_*) are equal to 0.5 and 7, respectively. For dynamic analysis with a strain rate of 1.25 × 10^−2^, compared with finite element model DFS4 (*λ* = 2.3), ring stiffness of finite element model DFS8 (*λ* = 4.0) decreases by 68.2% and 60.4%, when the displacement ductility coefficients (Δ/Δ*_y_*) are equal to 0.5 and 7, respectively. Regardless of the strain rate, the ring stiffness of the PPSRC beam–column joint decreases as the shear span-to-depth ratio increases. However, stiffness degradation of the PPSRC beam–column joint under a smaller value of *λ* is more remarkable than that under a larger value of *λ*. Another conclusion is given through Figure 12 that the initial stiffness of the PPSRC joint is higher under a larger strain rate. However, the stiffness degradation at a higher strain rate is more obvious under a larger displacement level. Another finding is that the ring stiffness under the reverse direction is smaller than that under the forward direction at each displacement level.

### 4.5. Ductility Performance

Ductility performance is the property that allows the PPSRC beam–column joint to undergo a large deformation beyond the initial yielding deformation without abruptly losing its bearing capacity. The ductility performance of the PPSRC beam–column joint can be represented by the displacement ductility factor (*μ*_∆_). The following formula will be adopted to calculate *μ*_∆_ [6].

(23) $$\mu_{\Delta }=\Delta_{u}/\Delta_{y}$$

where ∆*_u_* is ultimate displacement; ∆*_y_* is yielding displacement. ∆*_y_* in Equation (23) is obtained by the equivalent yield point determined on the skeleton curve. The “equivalent energy method” [41] is used to define an equivalent yielding point. The ultimate displacement ∆*_u_* in Equation (23) is obtained from the descending section of the skeleton curve where the bearing capacity is reduced to 85% of the maximum value. The calculation results of displacement ductility coefficients are shown in Table 5.

From Table 5, it can be observed that there are no obvious changes of displacement ductility coefficients for PPSRC joints with different strain rates. In other words, the effect of strain rate on ductility performance of the PPSRC joint is minimal. When referred to the shear span-to-depth ratio, regardless of strain rate, displacement ductility factor of the PPSRC beam–column joint increases gradually as the value of *λ* increases. That is to say, the ductility performance of the PPSRC beam–column joint with a larger value of *λ* is better than that with a smaller value of *λ*.

### 4.6. Energy Consumption

Another main concern in the dynamic behavior of the PPSRC joint is energy consumption. When estimating the energy consumption capacity of the PPSRC joint, eleven models are analyzed to obtain the variation law of cumulative energy consumption of the PPSRC joint with different strain rates or shear span-to-depth ratios. The first cycle result of cyclic loading is calculated to assess the energy consumption of the PPSRC joint.

**Figure 13:**
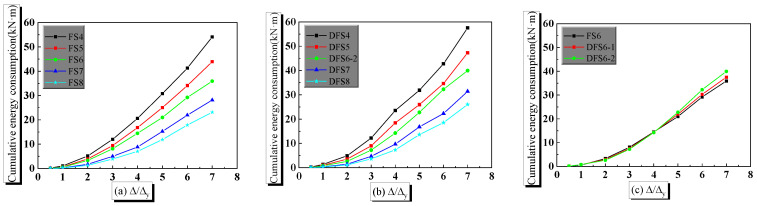
Curves of cumulative energy consumption for PPSRC joints: (**a**) models FS4, FS5, FS6, FS7 and FS8; (**b**) models DFS4, DFS5, DFS6-2, DFS7 and DFS8; (**c**) models FS6, DFS6-1 and DFS6-2.

It can be found from Figure 13, taking a displacement ductility coefficient equal to seven as an example, for quasi-static analysis with a strain rate of 1.25 × 10^−5^, the cumulative energy consumption of finite element models FS4 and FS8 are 54.1 kN·mm and 23.1 kN·mm, where the values of *λ* are 2.3 and 4.0, respectively. Compared with finite element model FS4, the cumulative energy consumption of finite element model FS8 decreases by 57.3%. For dynamic analysis with a strain rate of 1.25 × 10^−2^, the cumulative energy consumptions of finite element models DFS4 and DFS8 are 57.5 kN·mm and 26.0 kN·mm, where the values of *λ* are 2.3 and 4.0, respectively. Compared with finite element model DFS4, the cumulative energy consumption of finite element model DFS8 decreases by 54.8%. When the value of *λ* remains constant, compared with finite element model FS6, the cumulative energy consumption of finite element models DFS6-1 and DFS6-2 increase by 4.5% and 11.4%, respectively. Therefore, the cumulative energy consumption of the PPSRC beam–column joint is larger under a larger value of strain rate. Regardless of strain rate, the cumulative energy consumption of the PPSRC beam–column joint is smaller under a larger value of *λ*.

## 5. Reasonable Extension Length of Partial Steel Plate

As mentioned in the previous paper [19], the most severely damaged position of concrete is located at the end of the partial steel plate in the column, when the extension length of the partial steel plate is 370 mm. The concrete is crushed seriously, but the carrying capacity of the PPSRC joint does not decrease significantly. To improve the carrying capacity of the proposed PPSRC beam–column joint, an increased partial steel plate is needed. Based on the above considerations, the verified finite element model mentioned above is used through the software ABAQUS to calculate the reasonable extension length of the partial steel plate in the column. Previous studies show that the mechanical properties of the PPSRC beam–column joint are affected by the extension length of the partial steel plate in the column, the shear span-to-depth ratio and the strain rate. The influence extent of the strain rate is not remarkable, while the influence extent of the shear span-to-depth ratio is more obvious. Thus, for convenience of calculation, the strain rate effect is not included when calculating the reasonable extension length of the partial steel plate in the column.

**Figure 14:**
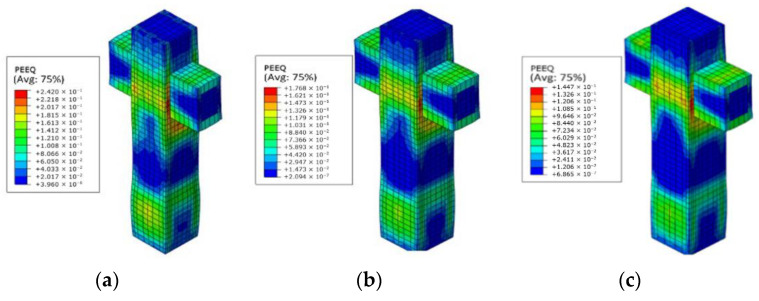

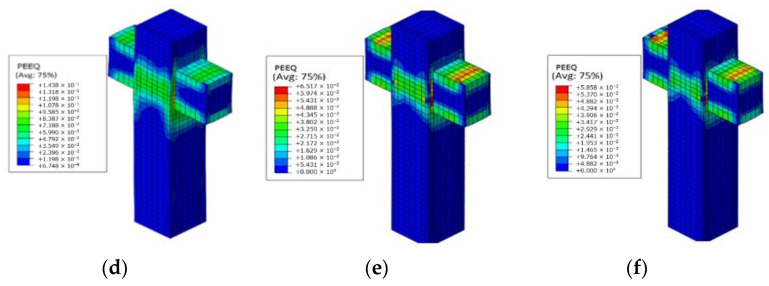
Contours of equivalent plastic strain under various values of *λ*: (**a**) *λ*=2.3; (**b**) *λ*=2.6; (**c**) *λ*=2.7; (**d**) *λ*=2.8; (**e**) *λ*=3.6; (**f**) *λ*=4.

To meet the existing specifications [20], the column should not be destroyed before the beam is destroyed. Contours of equivalent plastic strain were utilized to the study failure mode of the PPSRC beam–column joint, and the then reasonable extension length of partial steel plate in the column was determined according to the failure mode of the PPSRC beam–column joint. The value of *λ* increases gradually with a variation amplitude of 0.1, and failure modes of the PPSRC beam–column joint under various values of *λ* would be obtained. Taking an extension length of the partial steel plate in the column equal to 870 mm as an example, contours of equivalent plastic strain corresponding to several representative values of *λ* are given, which can be found in Figure 14.

With an increase in the value of *λ*, the concentration position of the equivalent plastic strain of the PPSRC beam–column joint also changes, and the equivalent plastic strain in the column decreases gradually. When the value of *λ* is smaller, concrete damage in the column is more serious; conversely, concrete damage in the beam and joint core area is more serious. Until the value of *λ* reaches 2.8, the equivalent plastic strain is concentrated in the beam and joint core area. Consequently, the reasonable extension length of partial steel plate in the column is 870 mm when the value of *λ* is greater than or equal to 2.8. Through further finite element analysis, the reasonable extension lengths of the partial steel plate corresponding to various values of *λ* are obtained, as given in Table 6.

## 6. Discussion and Conclusions

The main object of this is to extend the application of the PPSRC beam–column joint mentioned in the previous paper to the precast concrete structure. For this purpose, further analysis of the dynamic behavior of the PPSRC beam–column joint was carried out. In the light of the previous statement, relevant conclusions can be summarized.

(1) Ultimate bearing capacity and maximum displacement of numerical computation results are almost compatible with of the experimental results. Additionally, the changing trend of load-deflection hysteretic curves of numerical computation results is compatible with that of the experimental results. Moreover, the changing rule of equivalent plastic strain and concrete tensile damage of finite element analysis results is in good agreement with that of experimental results. Hence, the proposed finite element model can be utilized to assess the mechanical properties of the PPSRC beam–column joint.
(2) Both the yield carrying capacity and the ultimate carrying capacity of the PPSRC beam–column joint increase as the strain rate increases. Furthermore, regardless of the strain rate, the ring stiffness of the PPSRC beam–column joint decreases as the shear span-to-depth ratio increases. The ring stiffness under the reverse direction is smaller than that under the forward direction at each displacement level, and the initial stiffness of the PPSRC joint is higher under a larger strain rate. However, stiffness degradation with a higher strain rate is more obvious under a larger displacement level. Moreover, the ductility performance of the PPSRC beam–column joint is rarely affected by strain rate. However, regardless of the strain rate, the displacement ductility factor of the PPSRC beam–column joint increases gradually as the shear span-to-depth ratio increases. Finally, the energy consumption of the PPSRC beam–column joint increases as the strain rate increases. Compared with finite element model FS6, the cumulative energy consumption of finite element models DFS6-1 and DFS6-2 increase by 4.5% and 11.4%, respectively.
(3) Regardless of strain rate, the bearing capacity of the PPSRC beam–column joint decreases as the shear span-to-depth ratio increases. For quasi-static analysis or dynamic analysis, the ultimate bearing capacity of finite element model FS4 (λ = 2.3) is higher than that of finite element model FS8 (λ = 4.0). In addition, initial stiffness and ring stiffness of the PPSRC beam–column joints decrease as the shear span-to-depth ratio increases. However, stiffness degradation of the PPSRC beam–column joint is higher when the shear span-to-depth ratio is reduced. Moreover, the ductility performance of the PPSRC beam–column joint is better when the shear span-to-depth ratio is higher. Finally, the energy consumption of the PPSRC beam–column joint decreases when the shear span-to-depth ratio increases.
(4) The mechanical properties of the PPSRC beam–column joint are affected by the extension length of partial steel plate in the column, and the reasonable extension length of the partial steel plate in the column is related to the shear span-to-depth ratio. When the extension length of the partial steel plate is insufficient, the concrete at the end of the partial steel plate in the column is crushed seriously, but the carrying capacity of the PPSRC beam–column joint does not decrease significantly. When the extension length of the partial steel plate in the column is adequate, the concrete damage in the beam and joint core area is more serious.

## Figures and Tables

**Figure 6:**
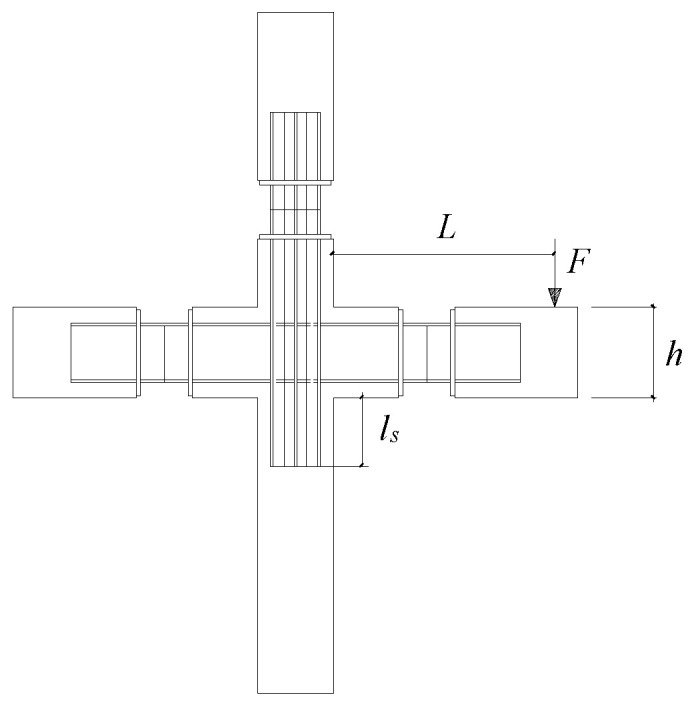
Diagrammatic sketch for each parameter.

**Table 1:** Material parameters of concrete.

*ρ* (kg/m^3^)	*μ*	*Ψ* (°)	*Ε’*	*α_f_*	*K*_1_	*ν*
2400	0.2	30	0.1	1.16	0.6667	0.005

**Table 2:** Measured results of material properties.

Material	Grade	Yielding Strength (MPa)	Ultimate Strength (MPa)	Elastic Modulus (GPa)
steel plate (12 mm)	Q235b	308	440	206
steel plate (20 mm)	Q235b	304	440	206
longitudinal reinforcement bar (18 mm)	HRB335	404	623	200
longitudinal reinforcement bar (22 mm)	HRB335	433	593	200
stirrup reinforcement (6 mm)	HPB235	335	475	210
stirrup reinforcement (8 mm)	HPB235	369	526	210
concrete	C30		22.4	31.1

Notes: The ultimate strength of concrete represents its prismatic compressive strength, and the dimension of the prismatic concrete specimen is 150 mm × 150 mm × 300 mm.

**Table 3:** General information of the finite element models.

Finite Element Models	Joint Type	Axial Compression Ratio	Shear Span-to-Depth Ratio	Steel Length in Column (mm)
FS2	interior beam–column joint	0.15	2.1	370
FSE1	exterior beam–column joint	0.1	2.3	370

Notes: The extension length of the partial steel plate in the column (*l_s_*) refers to the length of the steel plate under the joint core area. The shear span-to-depth ratio (*λ*) refers to the ratio of *L*/*h*, where *L* and *h* are the shear span length and effective beam height, respectively. The definition of each parameter can be found in Figure 6.

**Table 4:** Basic information of finite element analysis models.

Models	Joint Type	Shear Span-to-Depth Ratio	Strain Rate (1/s)	Axial Compression Ratio	Extension Length of Partial Steel Plate (mm)
FS4	interior PPSRC beam– column joint	2.3	1.25 × 10^−5^	0.05	870
FS5		2.6			
FS6		2.8			
FS7		3.6			
FS8		4.0			
DFS4		2.3	1.25 × 10^−2^	0.05	870
DFS5		2.6			
DFS7		3.6			
DFS8		4.0			
DFS6-1		2.8	1.25 × 10^−3^		
DFS6-2		2.8	1.25 × 10^−2^		

**Table 5:** Displacement ductility factors of PPSRC beam–column joints.

Displacement ductility coefficient	FS4	FS5	FS6	FS7
	2.69	2.78	2.85	3.10
	FS8	DFS4	DFS5	DFS7
	3.22	2.60	2.91	3.26
	DFS8	DFS6-1	DFS6-2	
	3.47	2.81	2.99	

**Table 6:** Reasonable extension length of the partial steel plate in the column.

*λ*	≥2.05	≥2.3	≥2.8
Reasonable extension length of partial steel plate in the column (mm)	1070	970	870

## Data Availability

The data presented in this study are available on request from the corresponding author.

## Nomenclature

*b* *_c_*	effective section width in *x* direction
*DIF* *_fc_*	dynamic increase factor
*d* *_c_*	effective section width in y direction
$d_{c}^{’}$	damage evolution coefficient of concrete under uniaxial compression
*d* *_t_*	damage evolution coefficient for concrete under uniaxial tension
*Ε’*	flow potential eccentricity
*E* *_c_*	elastic modulus for unconfined concrete
*f* *_c,r_*	a typical value corresponding to concrete strength under uniaxial compression
$f_{l,x}^{‘}$	effective restraint stresses in the *x*-direction
$f_{l,y}^{‘}$	effective restraint stresses in the *y*-direction
*f* *_t,r_*	a typical value corresponding to concrete strength under uniaxial tension
*f* *_x,h_*	yielding strength supplied by horizontal reinforcement bars
*f* *_y,h_*	yielding strength supplied by vertical reinforcement bars
HRC	highly restraint concrete
h	effective beam height
*K*	increasing factor of strength
*K*_1_	second stress invariant ratio of the compression meridian plane to the tension meridian plane
*K* *_c_*	stiffness of the descending stage
*K* *_e_*	stiffness of the elastic stage
$K_{e}^{’}$	effective restraint coefficient of stirrup reinforcements
$K_{j}^{i}$	ring stiffness
*K* *_s_*	stiffness of the post-yielding stage
L	shear span length
*l* *_s_*	extension length of the partial steel plate in the column
*n*	calculation parameter
PPSRC	precast and partial steel reinforced concrete
$P_{j}^{i}$	peak load under *i* cycles
PCC	partially confined concrete
PRC	partially restraint concrete
*P* *_y_*	yielding strength
$s^{\prime }$	vertical clear spacing of the spiral reinforcements or hooped reinforcements
UC	unconfined concrete
$w^{\prime }i$	clear spacing of the adjacent longitudinal reinforcements
*α* *_f_*	ultimate stress ratio of biaxial compression to uniaxial compression
*α* *_c_*	coefficient corresponding to descending branch of concrete stress–strain curve under uniaxial compression
*α* *_t_*	parameter value corresponding to the descending branch of concrete stress–strain curve under uniaxial tension
$\Delta_{j}^{i}$	peak displacement under i cycles
Δ*_B_*	peak displacement corresponding to the ultimate strength *P_u_*
∆*_u_*	ultimate displacement
Δ*_y_*	yielding displacement
Δ/Δ*_y_*	displacement ductility coefficient
*ε* *_c_*	strain under compression
$\varepsilon_{cc,hs}$	compressive strain corresponding to the concrete compressive strength of HRC
$\varepsilon_{cc,p}$	compressive strain corresponding to the concrete compressive strength of PCC
$\varepsilon_{cc,ps}$	compressive strain corresponding to the concrete compressive strength of PRC
$\varepsilon_{c,r}$	compressive strain corresponding to the concrete compressive strength of UC
*ε* *_c,r_*	compressive peak strain corresponding to the typical value of uniaxial compression strength *f_c,r_*
*ε* *_t_*	strain under tension
*ε* *_t,r_*	tensile peak strain corresponding to the typical value of uniaxial tension strength *f_t,r_*
*λ*	shear span-to-depth ratio
*μ*	Poisson ratio
*μ*_∆_	displacement ductility factor
*ν*	viscosity parameter
*ρ*	density
*ρ**_c_*,	calculation parameter
*ρ* *_cc_*	longitudinal reinforcement ratio
*ρ* *_t_*	calculation parameter
*ρ* *_x_*	reinforcement ratio of the rectangular reinforced concrete member in the horizontal direction
*ρ* *_y_*	reinforcement ratio of the rectangular reinforced concrete member in the vertical direction
*σ*	concrete stress
*σ* *_c_*	stress under compression
*σ* *_t_*	stress under tension
xc	calculation parameter
$x_{t}$	calculation parameter
*Ψ*	shear dilation angle
